# *De Novo* Assembly of the Donkey White Blood Cell Transcriptome and a Comparative Analysis of Phenotype-Associated Genes between Donkeys and Horses

**DOI:** 10.1371/journal.pone.0133258

**Published:** 2015-07-24

**Authors:** Feng-Yun Xie, Yu-Long Feng, Hong-Hui Wang, Yun-Feng Ma, Yang Yang, Yin-Chao Wang, Wei Shen, Qing-Jie Pan, Shen Yin, Yu-Jiang Sun, Jun-Yu Ma

**Affiliations:** 1 Institute of Reproductive Science, Qingdao Agricultural University, Qingdao, Shandong, 266109, China; 2 Key Laboratory of Animal Reproduction and Germplasm Enhancement in Universities of Shandong, Qingdao Agricultural University, Qingdao, Shandong, 266109, China; 3 College of Animal Science and Technology, Qingdao Agricultural University, Qingdao, Shandong, 266109, China; 4 Black Donkey Research Institute, Shandong Dongeejiao Company Limited, Liaocheng, Shandong, 252000, China; Chinese Academy of Fishery Sciences, CHINA

## Abstract

Prior to the mechanization of agriculture and labor-intensive tasks, humans used donkeys (*Equus africanus asinus*) for farm work and packing. However, as mechanization increased, donkeys have been increasingly raised for meat, milk, and fur in China. To maintain the development of the donkey industry, breeding programs should focus on traits related to these new uses. Compared to conventional marker-assisted breeding plans, genome- and transcriptome-based selection methods are more efficient and effective. To analyze the coding genes of the donkey genome, we assembled the transcriptome of donkey white blood cells *de novo*. Using transcriptomic deep-sequencing data, we identified 264,714 distinct donkey unigenes and predicted 38,949 protein fragments. We annotated the donkey unigenes by BLAST searches against the non-redundant (NR) protein database. We also compared the donkey protein sequences with those of the horse (*E*. *caballus*) and wild horse (*E*. *przewalskii*), and linked the donkey protein fragments with mammalian phenotypes. As the outer ear size of donkeys and horses are obviously different, we compared the outer ear size-associated proteins in donkeys and horses. We identified three ear size-associated proteins, HIC1, PRKRA, and KMT2A, with sequence differences among the donkey, horse, and wild horse loci. Since the donkey genome sequence has not been released, the *de novo* assembled donkey transcriptome is helpful for preliminary investigations of donkey cultivars and for genetic improvement.

## Introduction

For thousands of years of human history, donkeys were mainly breeded for farm labor or packing goods. In 2000, the world donkey population was estimated at approximately 43.5 million, but only 41 million donkeys existed in 2006 (a 5.7% reduction) [1]. China has the most donkeys, and its domestic donkey population increased from 7.4 million in 1966 to 10.923 million in 1996 [2]; however, based on the latest population survey, there were only 6.891 million donkeys in 2007 (a 37% reduction) [3]. The decreasing of donkey population in China is mainly attributed to agricultural mechanization, and partly to the slow speed of donkey breeding (single birth and long pregnancy). The accumulation of artificially selected characters important for farm labor over thousands of years are not suited to the modern demands in China for donkey meat, milk, and fur. Therefore, cultivating donkey breeds with new traits based on the demands of modern society is essential for the growth of the donkey industry.

In modern livestock breeding, genomes are important for cultivar characterization and genetic improvement. Conventional marker-assisted selection requires onerous phenotype data collection and its applications in commercial breeding have not generated desired results [4]. The cost of next-generation sequencing technology is sharply decreasing; accordingly, more livestock genomes have been released, and genome selection methods for livestock breeding have been developed [5]. In 2009, the first horse genome was released [6], and a horse-specific whole-genome single nucleotide polymorphism (SNP) chip was developed and successfully used for screening genes associated with Lavender Foal Syndrome [7]. Recently, it has been reported that a high-density horse genome SNP chip can be used to identify the genotypes of extant Perissodactyla, including donkeys [8]. However, as the horse SNP chip does not include all donkey SNPs, it cannot be directly applied to donkey breeding. In addition to a genome SNP chip, cDNA SNPs (cSNPs) or protein polymorphisms are more convenient for phenotype-genotype association analyses and for livestock breeding selection [9]. Livestock transcriptome sequencing can provide information not only regarding cSNP/protein polymorphism, but also about the expression levels of corresponding genes. As obtaining livestock cSNP data by transcriptome sequencing is faster and cheaper than obtaining genome SNP data, particularly for species without released genome sequences like the donkey, transcriptome analysis may helpful for the initial design of breeding plans.

To investigate protein sequence differences between donkeys and horses and to link donkey genotypes to phenotypes, we assembled the donkey white blood cell transcriptome *de novo*. By BLAST searching against the public non-redundant (NR) protein database, we annotated the donkey transcriptome. These results will be helpful for preliminary investigations of donkey genotype-phenotype associations. We also linked predicted donkey protein fragments with mammalian phenotypes [10]. As the outer ears of donkey are notably bigger than those of horses or wild horses, we analyzed the outer ear morphology-associated genes in the donkey, horse, and wild horse using the predicted donkey protein fragments. This association analysis improves our understanding of the phenotypic differences between donkeys and horses.

## Results

### *De novo* assembly of the donkey white blood cell transcriptome

**Fig 1:**
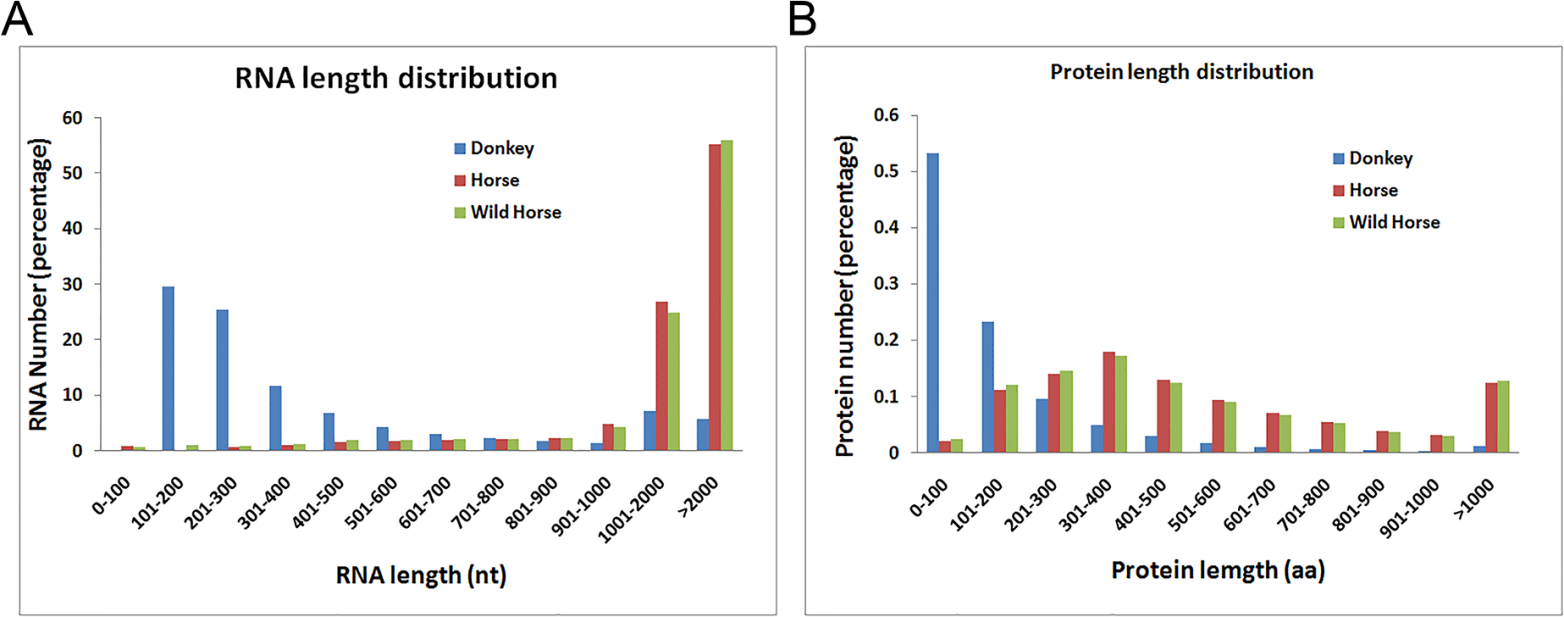
Length distribution of the *de novo* assembled donkey RNAs and predicted donkey protein fragments. The RNA and protein data for the horse and wild horse were downloaded from the NCBI genome database (ftp://ftp.ncbi.nih.gov/genomes).

Using high-throughput deep sequencing of the transcriptome of white blood cells sampled from the Dezhou donkey, we obtained approximately 129 million clean reads. We used these clean reads to assemble 358,946 unigenes (average length, 1,412 nt), of which 264,714 were distinct singletons. The average sequencing depth of these distinct singletons was 59 and the average GC content of the singletons was 44.77% (S1 Dataset). By aligning these singleton unigenes to the NR protein database using the blastx program, we predicted 38,949 unique donkey protein fragments (average length, 168 aa). As the donkey genome has not been fully sequenced and annotated, we compared the donkey unigenes and predicted proteins with data from the horse and wild horse. The length distributions of singleton unigenes and their corresponding predicted proteins are shown in Fig 1. The number and length of RNA fragments and proteins of the white blood cells of the donkey, horse, and wild horse are shown in Table 1. The total RNA length of *de novo* assembled donkey unigenes was 161.07% of the total RNA length of the horse and 122.20% of the total RNA length of the wild horse. However, the total protein length of predicted donkey protein fragments was only 35.27% of the total protein length of the horse and 29.55% of the total protein length of the wild horse, indicating that the *de novo* assembled donkey transcriptome includes a great many non-coding regions, such as untranslated regions, non-coding RNAs, or mis-assembled sequences. The assembled donkey unigenes and predicted protein fragments are listed in Supporting Information (S2 Dataset).

**Table 1:** Summary of the RNA/protein numbers and lengths of donkey white blood cells, horse, and wild horse.

Species (sample)	Donkey (*Equus africanus asinus*) white blood cells	Horse (*Equus caballus*)	Wild horse (*Equus przewalskii*)
RNA number	264,714	35,999	45,076
Average RNA length	594 nt	2,711 nt	2,854 nt
Total RNA length	157,214,590 nt	97,609,315 nt	128,651,487 nt
Protein number	38,949	32,352	38,416
Average protein length	168 aa	573 aa	575 aa
Total protein length	6,538,837 aa	18,540,986 aa	22,124,456 aa

### Analysis of predicted donkey proteins

To further analyze the predicted donkey proteins, we aligned them with horse and wild horse proteins by using the blastp program. As a result, 28,098 donkey protein fragments (72.14%) were aligned to 10,693 horse proteins (33.05%), and 28,180 donkey protein fragments (72.35%) were aligned to 11,213 wild horse proteins (29.20%). Based on the blast results, we found that the average identity between donkey proteins and horse and wild horse proteins were 94.32% and 94.12%, respectively, and the average positive sequence rates were 95.79% and 95.68%.

To determine associations between donkey protein fragments and mammalian phenotypes, we annotated donkey and horse proteins using the mammalian phenotype dataset from the Mouse Genome Informatics database (www.informatics.jax.org). As a result, 10,480 distinct donkey protein fragments were classified according to their associations with mammalian phenotypes (Table 2 and S3 Dataset). Although white blood cell mRNAs cannot be used to analyze associations between all coding regions and mammalian phenotypes, Table 2 shows that white blood cell mRNAs contain more than a half of the corresponding genes underlying mammalian phenotypes such as tumorigenesis (53.99%), liver/biliary system phenotypes (52.95%), hematopoietic system phenotypes (50.96%), embryogenesis (50.31%), and cellular phenotypes (50.37%).

**Table 2:** Statistical summary of predicted donkey protein fragments associated with mammalian phenotypes.

Mammalian Phenotype Term	Mouse symbol number	Mouse symbol number 2(percentage)	Horse protein number	Donkey protein fragment number	Donkey protein length (aa)	Donkey RNA length (nt)
adipose tissue phenotype	663	289 * (43.59%)	732	885	196,803	1,704,605
behavior/neurological phenotype	2,701	954 (35.32%)	2,765	3,012	624,409	5,874,119
cardiovascular system phenotype	2,053	893 (43.50%)	2,257	2,858	609,864	6,025,705
cellular phenotype	2,702	1361 (50.37%)	3,213	4,024	901,466	8,036,005
craniofacial phenotype	1,067	386 (36.18%)	1,024	1,274	285,326	2,325,180
digestive/alimentary phenotype	1,021	393 (38.49%)	1,077	1,138	215,507	1,830,532
embryogenesis phenotype	1,451	730 (50.31%)	1,694	2,099	512,414	4,358,464
endocrine/exocrine gland phenotype	1,636	705 (43.09%)	1,846	2,108	455,282	4,297,417
growth/size/body phenotype	3,649	1494 (40.94%)	3,787	4,644	1,076,374	9,211,980
hearing/vestibular/ear phenotype	595	186 (31.26%)	562	575	143,938	1,201,663
hematopoietic system phenotype	2,549	1299 (50.96%)	2,784	4,198	928,483	8,584,817
homeostasis/metabolism phenotype	3,649	1586 (43.46%)	3,977	5,096	1,046,174	9,943,604
immune system phenotype	2,604	1287 (49.42%)	2,873	4,269	927,245	8,861,768
integument phenotype	1,699	588 (34.61%)	1,541	1,911	413,608	3,736,364
limbs/digits/tail phenotype	986	298 (33.15%)	790	891	227,258	1,696,015
liver/biliary system phenotype	899	476 (52.95%)	1,055	1,318	298,347	2,662,206
mortality/aging	4,173	1903 (45.60%)	4,576	6,156	1,400,869	12,975,182
muscle phenotype	1,070	466 (43.55%)	1,305	1,456	309,052	3,014,111
nervous system phenotype	2,689	1068 (39.72%)	3,108	3,267	702,258	6,064,300
other phenotype	160	73 (45.63%)	173	248	43,176	551,780
pigmentation phenotype	582	150 (25.77%)	371	544	127,213	1,149,165
renal/urinary system phenotype	913	372 (40.74%)	972	1,195	265,898	2,162,417
reproductive system phenotype	1,649	658 (39.90%)	1,760	2,020	462,698	3,716,326
respiratory system phenotype	1,024	426 (41.60%)	1,127	1,251	256,160	2,294,374
skeleton phenotype	1,579	639 (40.47%)	1,620	1,996	428,884	3,722,388
taste/olfaction phenotype	111	37 (33.33%)	123	122	19,594	172,984
tumorigenesis	552	298 (53.99%)	627	1,177	247,409	1,907,737
vision/eye phenotype	1,274	497 (39.01%)	1,263	1,528	289,137	2,620,264

*Mouse symbol number 2 represents the number of mouse symbols whose donkey homologous protein fragments are detected in donkey white blood cells.

### Comparison of outer ear morphology-associated proteins between donkeys and horses

**Fig 2:**
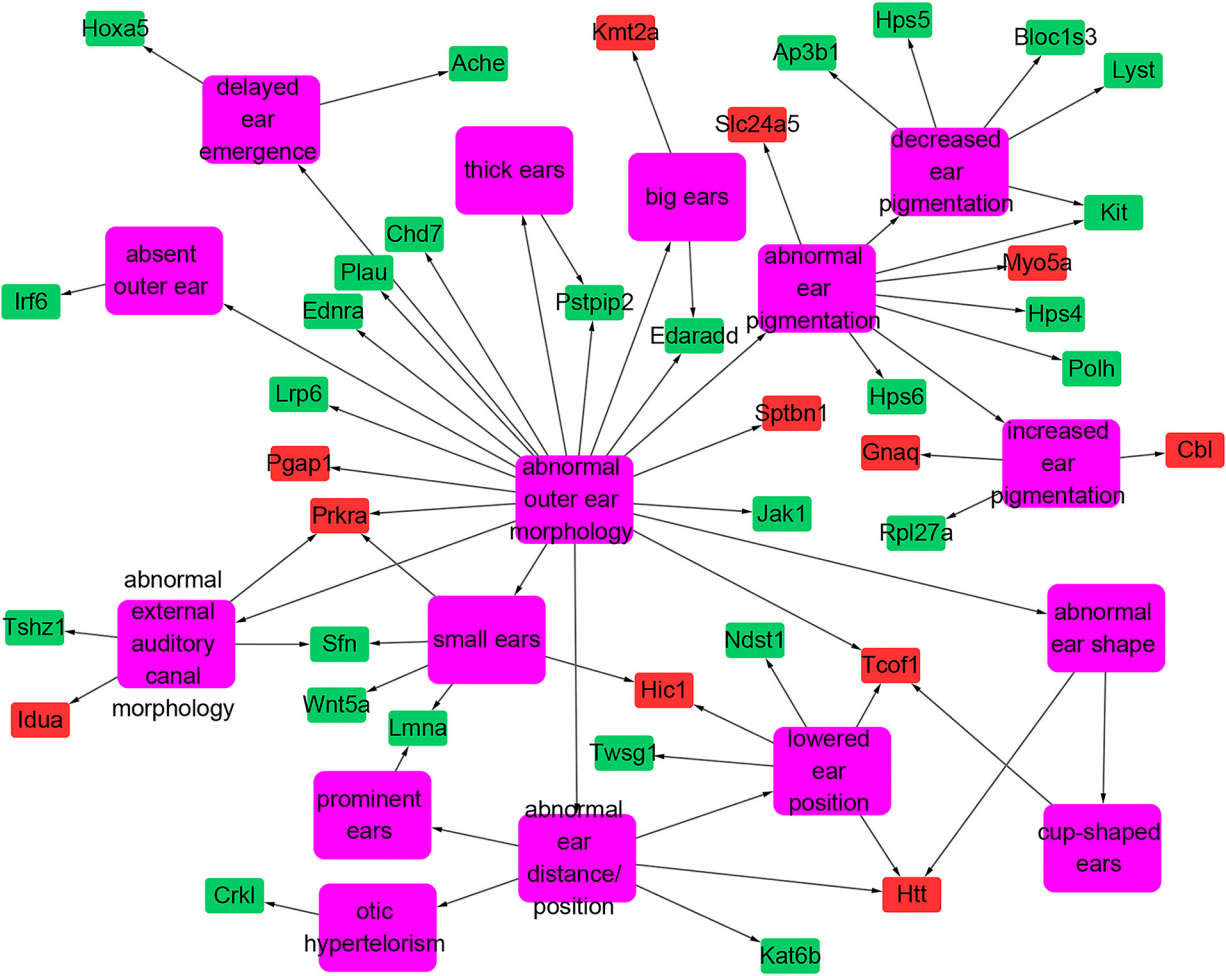
Outer ear morphology-associated genes. Only those genes whose homologous sequences were identified in the donkey white blood cell transcriptome are shown. Pink, mammalian phenotype term; green and red, mouse protein symbols associated with outer ear morphology. Red symbols represent the homologous donkey proteins that are not conserved between donkeys and horses.

**Fig 3:**
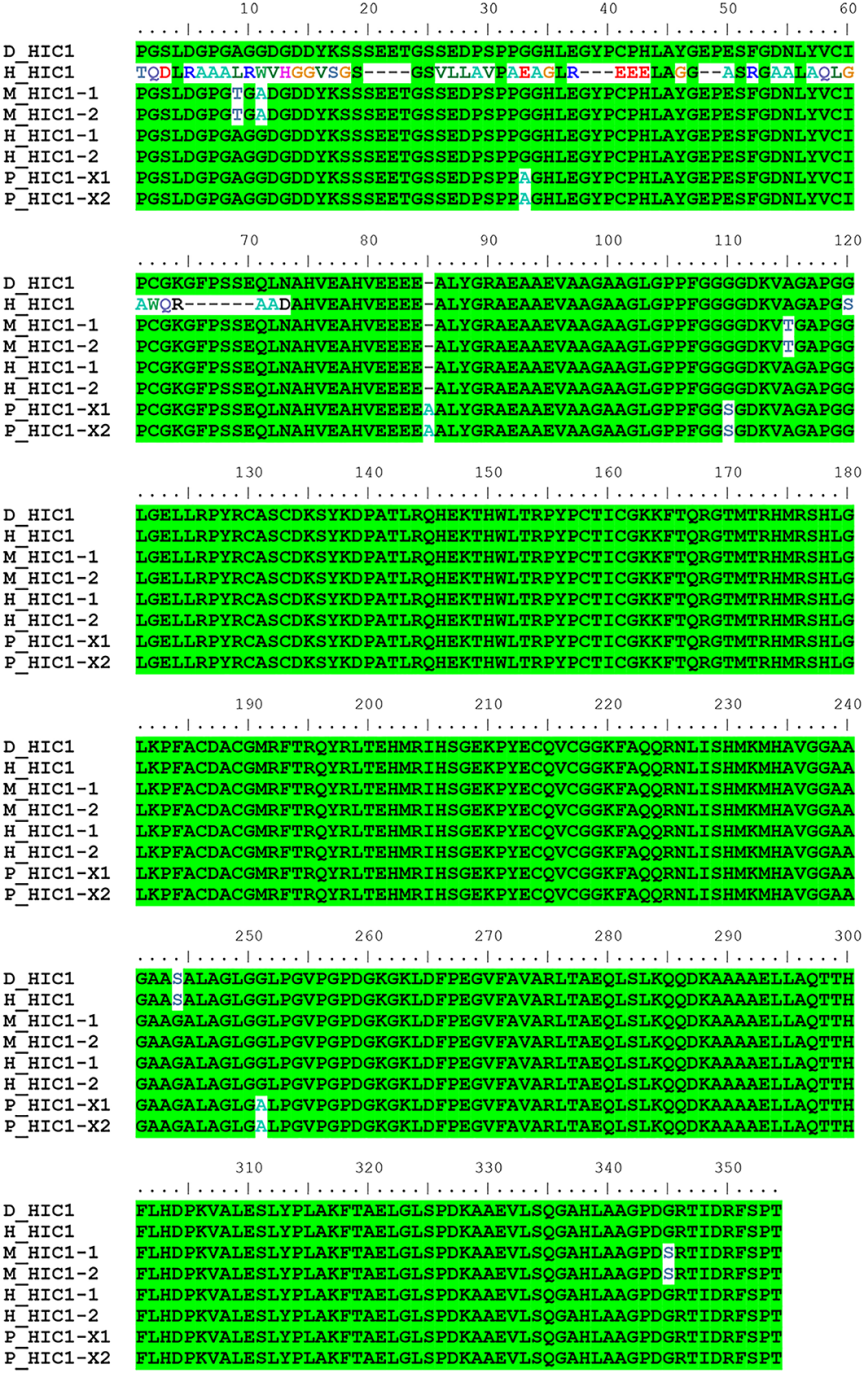
Multiple alignment of the HIC1 protein. The protein IDs and corresponding GenBank accession numbers are listed in Table 3.

**Fig 4:**
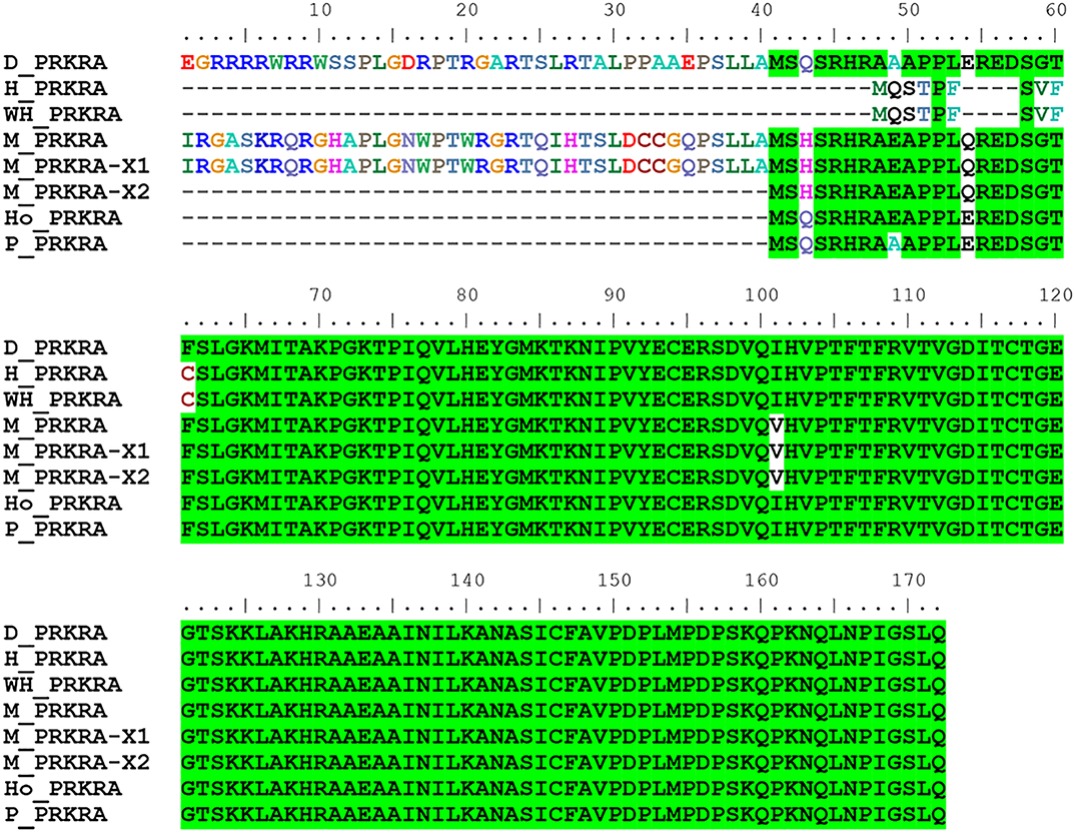
Multiple alignment of the PRKRA protein. The protein IDs and corresponding GenBank accession numbers are listed in Table 3.

As is commonly known, the outer ear of the donkey is longer than those of horses and wild horses. Using a mammalian phenotype database, we obtained 125 mouse genes that are associated with outer ear morphology. Based on BLAST searches of donkey, horse, and mouse proteins, we found 138 predicted donkey protein fragments (corresponding to 39 mouse proteins) associated with outer ear morphology (Fig 2). Three of these proteins (KMT2A, lysine-specific methyltransferase 2A; HIC1, hypermethylated in cancer 1; and PRKRA, protein kinase, interferon inducible double-stranded RNA dependent activator) were not conserved between horses and donkeys. We obtained multiple sequence alignments of the homologous proteins of the donkey, horse, wild horse, mouse, human, and pig. We found that the N-terminal region of the horse HIC1 protein (1–368 aa of GI: 545180679) was not conserved with those of the other species (partially displayed in Fig 3). We also found that horse and wild horse PRKRA proteins have the same 10 N-terminal amino acids, which cannot be aligned to the PRKRA locus of all other species including the donkey (Fig 4). Additionally, the horse KMT2A lacked the regions spanning amino acids 1304–1569 (region A) and 2038–2092 (region B) of donkey KMT2A. The lack of region A was horse-specific and the extra region B of donkey KMT2A was donkey-specific (S1 Fig).

## Discussion

As the donkey genome has not been published, the *de novo* assembled donkey white blood cell transcriptome is useful for preliminary investigations of associations between donkey genotypes and phenotypes. In our study, we predicted 6,538,837 amino acids of donkey protein sequences (accounting for 35% of the total protein length observed in horses). Using these predicted donkey proteins, we investigated the proteins that control donkey phenotypes, such as the outer ear size. We identified 3 outer ear size-associated proteins, HIC1, KMT2A, and PRKRA, and examined sequence differences between donkeys and horses/wild horses. HIC1 is a tumor suppressor protein that suppresses the overexpression of sirtuin 1 and maintains the activity of p53 to induce apoptosis of DNA-damaged cells [11]. HIC1-deficient mice show general developmental delay and underdeveloped outer ears [12]. The mutated N-terminal region of horse HIC1 includes the GLDLSKK motif, which mediates the transcriptional repression activity of HIC1 [13]. PRKRA is involved in cell apoptosis induced by its translation inhibition activity. It is also required for DICER1-mediated small interfering RNA production [14, 15]. In mouse embryos, *PRKRA* mRNA can be detected in the developing ear at embryonic day 12 [16]. In adult mice, PRKRA is expressed in all the regions of the pinna, middle ear, and cochlea [16]. When PRKRA is deleted, both ear development and auditory senses are impaired [16]. The N-terminal sequence variation between horse and wild horse PRKRA and other species indicates that transcription or translation differs from that of other species. KMT2A, a myeloid/mixed lymphoid leukemia gene, is a histone H3 lysine 4-specific methyltransferase. When fused with AF4/FMR2 family member proteins like AF4, KMT2A induces lymphoid and myeloid deregulation, and even induces hematologic malignancy [17]. In addition, KMT2A-AF4 induces body growth retardation and an abnormally large outer ear [17]. We found that horse KMT2A (gi|545221883) lacks 266-aa-long fragments corresponding to region A of donkey KMT2A. However, other versions of horse KMT2A (Uniprot: F6U6A9_HORSE) and wild horse KMT2A do not lack the 266-aa fragment, indicating a sequencing error or differences among breeds.

In this study, we developed a workflow to link donkey protein sequences with mammalian phenotypes, and compared phenotype-associated proteins between donkey and horse accessions. This workflow is helpful for analyzing functional genes in donkeys, and it will improve donkey breeding. Although protein differences cannot fully explain the phenotype differences among donkeys, horses, and wild horses, our data can be used to preliminarily explore differences in body size and other traits important for human breeding programs. In addition, there are some limitations in our study. Firstly, a gene mutation which induces a phenotype in mice may not necessarily induce the same phenotype in donkeys; secondly, as the *de novo* assembled genes cannot contain all of the associated genes of a phenotype, our data can only be used as a reference resource for donkey phenotype analysis.

In summary, we assembled the donkey white blood cell transcriptome *de novo* and linked donkey unigenes to mammalian phenotypes. We will further investigate cSNP selection methods in donkeys and identify gene markers associated with body size, milk production, and dermal thickness.

## Materials and Methods

### Ethics Statement

The donkey blood isolation manipulations were performed according to the suggestions in the Guideline for Animal Experimentation in Qingdao Agricultural University. Donkey jugular vein blood (5–8 ml) was collected using disposable vacuum blood collection tubes and blood-taking needles. All animal manipulations were performed under the approval of the Animal Care and Ethics Committee of Qingdao Agricultural University.

### Preparation donkey white blood cell samples

The white blood cells used for deep transcriptome sequencing were isolated from a female Dezhou donkey from the Black Donkey Research Institute (Liaocheng, Shandong, China) using a peripheral blood white cell separation kit (WBC1094, TBD Science, Tianjin, China). The isolated white cells were stored in RNAlater Stabilization Solution (AM7020, Life Technologies) and sent to the Beijing Genome Institute (BGI-Shenzhen) for transcriptome library construction and RNA-seq.

### Deep transcriptome sequencing of donkey white blood cells

All deep transcriptome sequencing procedures were performed at the Beijing Genome Institute (Shenzhen, China). Briefly, total RNAs were isolated and mRNAs were extracted by magnetic beads with Oligo(dT). These mRNAs were fragmented and used as templates to synthesize cDNAs. After purification and single nucleotide adenine addition, short fragments were linked to adapters for amplification. The amplified library was sequenced using the Illumina Hiseq 2000 platform. The raw sequencing data in fastq format was uploaded to the Sequence Read Archive (SRA) under the accession number SRX973470.

### RNA-seq data processing and donkey transcriptome annotation

The read adaptors and low-quality reads from raw RNA-seq reads were removed, and the *de novo* assembly was carried out with the RNA-seq *de novo* assembly software Trinity [18]. The new assembled donkey unigenes were annotated and the corresponding protein fragments were predicted using BAST searches of the unigene sequence to the non-redundant (NR) protein database.

### Comparative analysis of RNA and protein sequences among the donkey, horse, and wild horse

The protein data for the mouse, horse [6], and wild horse [19] were downloaded from the NCBI genome database (ftp://ftp.ncbi.nih.gov/genomes). The number and length of RNA and protein fragments were summarized using a python script. The predicted donkey unigene proteins were aligned to the horse or wild horse protein dataset using the blastp program. Homologous sequences among the donkey, horse, and wild horse were identified by applying an identity threshold of 80% [20].

To link the donkey predicted protein fragments to mammalian phenotypes, mammalian phenotype data was extracted from the Mammalian Phenotype Browser (www.informatics.jax.org) [10]. As the predicted donkey protein fragments were not integrated, horse proteins were used to query homologous proteins between the mouse and *Equus* species. The mouse protein sequences were compared with those in horses, and the most similar sequences with identities of greater than 80% were considered homologous.

### Analysis of *Equus* outer ear morphology-associated proteins

The information regarding genes associated with outer ear morphology (MP: 0002177) was extracted from the Mammalian Phenotype Browser [10]. The outer ear morphology-associated proteins that had no homology to horse genes or donkey sequences identified in the *de novo* assembled transcriptome were ignored. The donkey protein fragments that were not fully aligned to homologous horse proteins and whose protein identities were less than 98%, were analyzed to determine the genotype controlling *Equus* outer ear morphology. To analyze the outer ear size associated-proteins, outer ear size associated-protein sequences of the mouse, human, and pig were obtained, and a multiple sequence alignment was generated and analyzed (Table 3).

**Table 3:** Protein sequences used for multiple sequence alignment.

Symbol / Full name	Abbreviation	Species	ID
HIC1 / hypermethylated in cancer 1	D_HIC1	Donkey	Unigene110778_NormalA
	H_HIC1	Horse	gi|545180679
	M_HIC1-1	Mouse	gi|148226885
	M_HIC1-2	Mouse	gi|148228529
	H_HIC1-1	Human	gi|61676186
	H_HIC1-2	Human	gi|148237270
	P_HIC1-X1	Pig	gi|311268073
	P_HIC1-X2	Pig	gi|545859615
PRKRA / protein kinase, interferon inducible double stranded RNA dependent activator	D_PRKRA	Donkey	Unigene69853_NormalA
	H_PRKRA	Horse	gi|545191414
	WH_PRKRA	Wild Horse	gi|664713499
	M_PRKRA	Mouse	gi|755499568
	M_PRKRA-X1	Mouse	gi|755499566
	M_PRKRA-X2	Mouse	gi|6755162
	Ho_PRKRA	Human	gi|49065476
	P_PRKRA	Pig	gi|194043952
KMT2A / lysine-specific methyltransferase 2A	D_KMT2A	Donkey	Unigene1721_NormalA
	H_KMT2A	Horse	gi|545221883
	WH_KMT2A	Wild Horse	gi|664709525
	M_KMT2A	Mouse	gi|124486682
	H_KMT2A-1	Human	gi|308199413
	H_KMT2A-2	Human	gi|56550039
	P_KMT2A	Pig	gi|350588548

Cytoscape was used to visualize the gene–phenotype associations [21]. Python scripts were written to extract and analyze data and these scripts can be obtained on requested.

## Supporting Information

S1 Fig Multiple alignment of the KMT2A protein. The protein IDs and corresponding GenBank accession numbers are listed in Table 3.
(PDF)

S1 Dataset Lengths and GC percents of assembled donkey genes. (ZIP)

S2 Dataset Assembled donkey genes and predicted proteins. (ZIP)

S3 Dataset Phenotype annotation of donkey assembled genes. (XLSX)
