# Supplementary material for: De Novo Assembly of the Donkey White Blood Cell Transcriptome and a Comparative Analysis of Phenotype-Associated Genes between Donkeys and Horses
Source: PLoS One. 2015 Jul 24;10(7):e0133258. doi: 10.1371/journal.pone.0133258 (PMC4514889; doi:10.1371/journal.pone.0133258)
Supplement: S1 Fig — The protein IDs and corresponding GenBank accession numbers are listed in Table 3. (PDF) [file pone.0133258.s001.pdf]

```
                     10        20        30        40        50        60        70        80
             ....|....|....|....|....|....|....|....|....|....|....|....|....|....|....|....|
D_KMT2A      DIVKIIQAAINSDGGQPEIKKANSMVKSFFIRQMERVFPWFSVKKSRFWEPNKVSSNSGMLPNAVLPPSLDHNYAQWQER
H_KMT2A      DIVKIIQAAINSDGGQPEIKKANSMVKSFFIRQMERVFPWFSVKKSRFWEPNKVSSNSGMLPNAVLPPSLDHNYAQWQER
WH_KMT2A     DIVKIIQAAINSDGGQPEIKKANSMVKSFFIRQMERVFPWFSVKKSRFWEPNKVSSNSGMLPNAVLPPSLDHNYAQWQER
M_KMT2A      DIVKIIQAAINSDGGQPEIKKANSMVKSFFIRQMERVFPWFSVKKSRFWEPNKVSNNSGMLPNAVLPPSLDHNYAQWQER
H_KMT2A-1    DIVKIIQAAINSDGGQPEIKKANSMVKSFFIRQMERVFPWFSVKKSRFWEPNKVSSNSGMLPNAVLPPSLDHNYAQWQER
H_KMT2A-2    DIVKIIQAAINSDGGQPEIKKANSMVKSFFIRQMERVFPWFSVKKSRFWEPNKVSSNSGMLPNAVLPPSLDHNYAQWQER
P_KMT2A      DIVKIIQAAINSDGGQPEIKKANSMVKSFFIRQMERVFPWFSVKKSRFWEPNKVSSNSGMLPNAVLPPSLDHNYAQWQER

                     90       100       110       120       130       140       150       160
             ....|....|....|....|....|....|....|....|....|....|....|....|....|....|....|....|
D_KMT2A      EEDSHTEQPPLMKKIIPAPKPKGPGEPDSPTPPHPPTPPILSTDRSREDSPELNPPPGIEDNRQCALCLTYGDDSANDAG
H_KMT2A      EEDSHTEQPPLMKKIIPAPKPKGPGEPDSPTPPHPPTPPILSTDRSREDSPELNPPPGIEDNRQCALCLTYGDDSANDAG
WH_KMT2A     EEDSHTEQPPLMKKIIPAPKPKGPGEPDSPTPPHPPTPPILSTDRSREDSPELNPPPGIEDNRQCALCLTYGDDSANDAG
M_KMT2A      EESSHTEQPPLMKKIIPAPKPKGPGEPDSPTPLHPPTPPILSTDRSREDSPELNPPPGIDDNRQCALCLMYGDDSANDAG
H_KMT2A-1    EENSHTEQPPLMKKIIPAPKPKGPGEPDSPTPLHPPTPPILSTDRSREDSPELNPPPGIEDNRQCALCLTYGDDSANDAG
H_KMT2A-2    EENSHTEQPPLMKKIIPAPKPKGPGEPDSPTPLHPPTPPILSTDRSREDSPELNPPPGIEDNRQCALCLTYGDDSANDAG
P_KMT2A      EENSHTEQPPLMKKIIPAPKPKGPGEPDSPTPLHPPTPPILSTDRSREDSPELNPPPGIEDNRQCALCLTYGDDSANDAG

                    170       180       190       200       210       220       230       240
             ....|....|....|....|....|....|....|....|....|....|....|....|....|....|....|....|
D_KMT2A      RLLYIGQNEWTHVNCALWSAEVFEDDDGSLKNVHMAVIRGKQLRCEFCQKPGATVGCCLTSCTSNYHFMCSRAKNCVFLD
H_KMT2A      RLLYIGQNEWTHVNCALWSAEVFEDDDGSLKNVHMAVIRGKQLRCEFCQKPGATVGCCLTSCTSNYHFMCSRAKNCVFLD
WH_KMT2A     RLLYIGQNEWTHVNCALWSAEVFEDDDGSLKNVHMAVIRGKQLRCEFCQKPGATVGCCLTSCTSNYHFMCSRAKNCVFLD
M_KMT2A      RLLYIGQNEWTHVNCALWSAEVFEDDDGSLKNVHMAVIRGKQLRCEFCQKPGATVGCCLTSCTSNYHFMCSRAKNCVFLD
H_KMT2A-1    RLLYIGQNEWTHVNCALWSAEVFEDDDGSLKNVHMAVIRGKQLRCEFCQKPGATVGCCLTSCTSNYHFMCSRAKNCVFLD
H_KMT2A-2    RLLYIGQNEWTHVNCALWSAEVFEDDDGSLKNVHMAVIRGKQLRCEFCQKPGATVGCCLTSCTSNYHFMCSRAKNCVFLD
P_KMT2A      RLLYIGQNEWTHVNCALWSAEVFEDDDGSLKNVHMAVIRGKQLRCEFCQKPGATVGCCLTSCTSNYHFMCSRAKNCVFLD

                    250       260       270       280       290       300       310       320
             ....|....|....|....|....|....|....|....|....|....|....|....|....|....|....|....|
D_KMT2A      DKKVYCQRHRDLIKGEVVPENGFEVFRRVFVDFEGISLRRKFLNGLEPENIHMMIGSMTIDCLGILNDLSDCEDKLFPIG
H_KMT2A      DKKVYCQRHRDLIKGEVVPENGFEVFRRVFVDFEGISLRRKFLNGLEPENIHMMIGSMTIDCLGILNDLSDCEDKLFPIG
WH_KMT2A     DKKVYCQRHRDLIKGEVVPENGFEVFRRVFVDFEGISLRRKFLNGLEPENIHMMIGSMTIDCLGILNDLSDCEDKLFPIG
M_KMT2A      DKKVYCQRHRDLIKGEVVPENGFEVFRRVFVDFEGISLRRKFLNGLEPENIHMMIGSMTIDCLGILNDLSDCEDKLFPIG
H_KMT2A-1    DKKVYCQRHRDLIKGEVVPENGFEVFRRVFVDFEGISLRRKFLNGLEPENIHMMIGSMTIDCLGILNDLSDCEDKLFPIG
H_KMT2A-2    DKKVYCQRHRDLIKGEVVPENGFEVFRRVFVDFEGISLRRKFLNGLEPENIHMMIGSMTIDCLGILNDLSDCEDKLFPIG
P_KMT2A      DKKVYCQRHRDLIKGEVVPENGFEVFRRVFVDFEGISLRRKFLNGLEPENIHMMIGSMTIDCLGILNDLSDCEDKLFPIG

                    330       340       350       360       370       380       390       400
             ....|....|....|....|....|....|....|....|....|....|....|....|....|....|....|....|
D_KMT2A      YQCSRVYWSTTDARKRCVYTCKIVECRPPVVEPDINSTVEHDENRTIAHSPSSLAEISSKESHNTAENISPPSPDRPPHS
H_KMT2A      YQCSRVYWSTTDARKRCVYTCKIVECRPPVVEPDINSTVEHDENRTIAHSPSSLAEISSKESHNTAENISPPSPDRPPHS
WH_KMT2A     YQCSRVYWSTTDARKRCVYTCKIVECRPPVVEPDINSTVEHDENRTIAHSPSSLAEISSKESHNTAENISPPSPDRPPHS
M_KMT2A      YQCSRVYWSTTDARKRCVYTCKIMECRPPVVEPDINSTVEHDDNRTIAHSPSSFIDASCKDSQSTAAILSPPSPDRP-HS
H_KMT2A-1    YQCSRVYWSTTDARKRCVYTCKIVECRPPVVEPDINSTVEHDENRTIAHSPTSFTESSSKESQNTAEIISPPSPDRPPHS
H_KMT2A-2    YQCSRVYWSTTDARKRCVYTCKIVECRPPVVEPDINSTVEHDENRTIAHSPTSFTESSSKESQNTAEIISPPSPDRPPHS
P_KMT2A      YQCSRVYWSTTDARKRCVYTCKIVECRPPVVEPDINSTVEHDENRTIAHSPSSFAEISSKESQNTAEIVSPPSPDRPPHS

                    410       420       430       440       450       460       470       480
             ....|....|....|....|....|....|....|....|....|....|....|....|....|....|....|....|
D_KMT2A      QTSGSCFYHVISKVPRIPTPSYSPTQRAPGCRPLPSAGSPTPTTHEIVTVGDPLLSSGLRSIGSRRHSTSSLSPQRSKLR
H_KMT2A      QTSGSCFYHVISKVPRIPTPSYSPTQRAPGCRPLPSAGSPTPTTHEIVTVGDPLLSSGLRSIGSRRHSTSSLSPQRSKLR
WH_KMT2A     QTSGSCFYHVISKVPRIPTPSYSPTQRAPGCRPLPSAGSPTPTTHEIVTVGDPLLSSGLRSIGSRRHSTSSLSPQRSKLR
M_KMT2A      QTSGSCYYHVISKVPRIRTPSYSPTQRSPGCRPLPSAGSPTPTTHEIVTVGDPLLSSGLRSIGSRRHSTSSLSPLRSKLR
H_KMT2A-1    QTSGSCYYHVISKVPRIRTPSYSPTQRSPGCRPLPSAGSPTPTTHEIVTVGDPLLSSGLRSIGSRRHSTSSLSPQRSKLR
H_KMT2A-2    QTSGSCYYHVISKVPRIRTPSYSPTQRSPGCRPLPSAGSPTPTTHEIVTVGDPLLSSGLRSIGSRRHSTSSLSPQRSKLR
P_KMT2A      QTSGSCFYHVISKVPRIRTPSYSPTQRSPGCRPLPSAGSPTPTTHEIVTVGDPLLSSGLRSIGSRRHSTSSLSPQRSKLR

                    490       500       510       520       530       540       550       560
             ....|....|....|....|....|....|....|....|....|....|....|....|....|....|....|....|
D_KMT2A      IMSPMRTGSTYSRNSVSSVSTVGTATDLESSTKAVDHVLGPLNSNTNLGQNTPTSSNLQRTVVTMGTKTSHLDGSSSSEM
H_KMT2A      IMSPMRTGSTYSRNSVSSVSTVGTATDLESSTKAVDHVLGPLNSNTNLGQNTPTSSNLQRTVVTMGTKTSHLDGSSSSEM
WH_KMT2A     IMSPMRTGSTYSRNSVSSVSTVGTATDLESSTKAVDHVLGPLNSNTNLGQNTPTSSNLQRTVVTMGTKTSHLDGSSSSEM
M_KMT2A      IMSPVRTGSAYSRSSVSSVPSLGTATDPEASAKASDRG-GLLSSSANLGHSAPPSSSSQRTVG--GSKTSHLDGSSPSEV
H_KMT2A-1    IMSPMRTGNTYSRNNVSSVSTTGTATDLESSAKVVDHVLGPLNSSTSLGQNTSTSSNLQRTVVTVGNKNSHLDGSSSSEM
H_KMT2A-2    IMSPMRTGNTYSRNNVSSVSTTGTATDLESSAKVVDHVLGPLNSSTSLGQNTSTSSNLQRTVVTVGNKNSHLDGSSSSEM
P_KMT2A      IMSPMRTGGTYSRNSVSSLSTIGTTTDLESSAKAADHVLGPLNSNTNLGQNTPTSSNLQRTVVTLGTKTSHLDGPSSSEM

                    570       580       590       600       610       620       630       640
             ....|....|....|....|....|....|....|....|....|....|....|....|....|....|....|....|
D_KMT2A      KHSTASDLASKSSSLKGEKTKMLSSKSSEGSAHNVAYPGIPKLAPQVHNTTSGELNVSKISTYVEHSSAPFSSKETLSFP
H_KMT2A      KHSTASDLASKSSSLKGEKTKMLSSKSSEGSAHNVAYPGIPKLAPQVHNTTSGELNVSKISTYVEHSSAPFSSKETLSFP
WH_KMT2A     KHSTASDLASKSSSLKGEKTKMLSSKSSEGSAHNVAYPGIPKLAPQVHNTTSGELNVSKISTYVEHSSAPFSSKETLSFP
```

```
M_KMT2A      KRCSASDLVPKGSLVKGEKNRTSSSKSTDGSAHSTAYPGIPKLTPQVHNATPGELNISKIGSFAEPSTVPFSSKDTVSYP
H_KMT2A-1    KQSSASDLVSKSSSLKGEKTKVLSSKSSEGSAHNVAYPGIPKLAPQVHNTTSRELNVSKIGSFAEPSSVSFSSKEALSFP
H_KMT2A-2    KQSSASDLVSKSSSLKGEKTKVLSSKSSEGSAHNVAYPGIPKLAPQVHNTTSRELNVSKIGSFAEPSSVSFSSKEALSFP
P_KMT2A      KHSSASDSTSKSSSLKGEKTKMPSSKNSEGSAHNVAYPGIPKLAPQVHNTTSGELNVSKMGTFAELSSVPFSSKEALPFP

                      650       660       670       680       690       700       710       720
             ....|....|....|....|....|....|....|....|....|....|....|....|....|....|....|....|
D_KMT2A      PVHLRGQRNDRDQHTDSNQSANPPPSEDTEVKTLKLSGVSNRSSITNEHVGSSSRDRRQKGKKSSKETFKEKHSSKSFLE
H_KMT2A      PVHLRGQRNDRDQHTDSNQSANPPPSEDTEVKTLKLSGVSNRSSITNEHVGSSSRDRRQKGKKSSKETFKEKHSSKSFLE
WH_KMT2A     PVHLRGQRNDRDQHTDSNQSANPPPSEDTEVKTLKLSGVSNRSSITNEHVGSSSRDRRQKGKKSSKETFKEKHSSKSFLE
M_KMT2A      QLHLRGQRSDRDQHMDPSQSVKPSPNEDGEIKTLKLPGMGHRPSILHEHIGSSSRDRRQKGKKSSKETCKEKHSSKSYLE
H_KMT2A-1    HLHLRGQRNDRDQHTDSTQSANSSPDEDTEVKTLKLSGMSNRSSIINEHMGSSSRDRRQKGKKSCKETFKEKHSSKSFLE
H_KMT2A-2    HLHLRGQRNDRDQHTDSTQSANSSPDEDTEVKTLKLSGMSNRSSIINEHMGSSSRDRRQKGKKSCKETFKEKHSSKSFLE
P_KMT2A      PLHLRGQRNDRDQQPDSNQLANPPPDEDTEVKTLKLSGVSSRASTISEHVGSSSRDRRQKGKKSGKDTFKEKHSSKSFLE

                      730       740       750       760       770       780       790       800
             ....|....|....|....|....|....|....|....|....|....|....|....|....|....|....|....|
D_KMT2A      PGQVTTGEEGNLKPESVDEVLTPEFMG-RPCNNVSSDKIGDKALSVPGVPKAPSMQVEGSAKELQTPRKRTVKVTLTPLK
H_KMT2A      PGQVTTGEEGNLKPESVDEVLTPEFMG-RPCNNVSSDKIGDKVLSIPGVPKAPSLQVEGSAKELQTPRKRTVKVTLTPLK
WH_KMT2A     PGQVTTGEEGNLKPESVDEVLTPEFMG-RPCNNVSSDKIGDKVLSIPGVPKAPSMQVEGSAKELQTPRKRTVKVTLTPLK
M_KMT2A      PGQVTTGEEGNLKPEFADEVLTPGFLGQRPCNNVSSEKIGDKVLPLSGVPKGQSTQVEGSSKELQAPRKCSVKVT--PLK
H_KMT2A-1    PGQVTTGEEGNLKPEFMDEVLTPEYMGQRPCNNVSSDKIGDKGLSMPGVPKAPPMQVEGSAKELQAPRKRTVKVTLTPLK
H_KMT2A-2    PGQVTTGEEGNLKPEFMDEVLTPEYMGQRPCNNVSSDKIGDKGLSMPGVPKAPPMQVEGSAKELQAPRKRTVKVTLTPLK
P_KMT2A      PGQVTTGEEGNLKPEFMDEVLTPEFMGQRPCNNVSSDKIGDKVHSIAGVPKAASMQVEGSAKELQTPRKRTVKVTLTPLK

                      810       820       830       840       850       860       870       880
             ....|....|....|....|....|....|....|....|....|....|....|....|....|....|....|....|
D_KMT2A      MESESQSKNTLKESSPVSPLQIESASPTEPISASESPGDGPVAQPSPNNTSSQDSQSNNYQNLPVQDRNLMLPDGPKPQE
H_KMT2A      MESESQSKNTLKESSPVSPLQIESASPTEPISASESPGDGPVAQPSPNNTSSQDSQSNNYQNLPVQDRNLMLPDGPKPQE
WH_KMT2A     MESESQSKNTLKESSPVSPLQIESASPTEPISASESPGDGPVAQPSPNNTSSQDSQSNNYQNLPVQDRNLMLPDGPKPQE
M_KMT2A      MEGENQSKNTQKESGPGSPAHIESVCPAEPVSASRSPGAGPGVQPSPNNTLSQDPQSNNYQNLPEQDRNLMIPDGPKPQE
H_KMT2A-1    MENESQSKNALKESSPASPLQIESTSPTEPISASENPGDGPVAQPSPNNTSCQDSQSNNYQNLPVQDRNLMLPDGPKPQE
H_KMT2A-2    MENESQSKNALKESSPASPLQIESTSPTEPISASENPGDGPVAQPSPNNTSCQDSQSNNYQNLPVQDRNLMLPDGPKPQE
P_KMT2A      MESESQSKNTLKESSPVSPLQIEPASPTEPVSASESPGDGPVAQPSPNNTSSQDSQSNSYQNLPVQDRNLMLPDGPKPQE

                      890       900       910       920       930       940       950       960
             ....|....|....|....|....|....|....|....|....|....|....|....|....|....|....|....|
D_KMT2A      DGSFKRRYPRRSARARSNMFFGLTPLYGVRSYGEEDIPFYSSSTGKKRGKRSAEGQVDGADDLSTSDEDDLYYYNFTRTV
H_KMT2A      DGSFKRRYPRRSARARSNMFFGLTPLYGVRSYGEEDIPFYSSSTGKKRGKRSAEGQVDGADDLSTSDEDDLYYYNFTRTV
WH_KMT2A     DGSFKRRYPRRSARARSNMFFGLTPLYGVRSYGEEDIPFYSSSTGKKRGKRSAEGQVDGADDLSTSDEDDLYYYNFTRTV
M_KMT2A      DGSFKRRYPRRSARARSNMFFGLTPLYGVRSYGEEDIPFYSNSTGKKRGKRSAEGQVDGADDLSTSDEDDLYYYNFTRTV
H_KMT2A-1    DGSFKRRYPRRSARARSNMFFGLTPLYGVRSYGEEDIPFYSSSTGKKRGKRSAEGQVDGADDLSTSDEDDLYYYNFTRTV
H_KMT2A-2    DGSFKRRYPRRSARARSNMFFGLTPLYGVRSYGEEDIPFYSSSTGKKRGKRSAEGQVDGADDLSTSDEDDLYYYNFTRTV
P_KMT2A      DGSFKRRYPRRSARARSNMFFGLTPLYGVRSYGEEDIPFYSSSTGKKRGKRSAEGQVDGADDLSTSDEDDLYYYNFTRTV

                      970       980       990      1000      1010      1020      1030      1040
             ....|....|....|....|....|....|....|....|....|....|....|....|....|....|....|....|
D_KMT2A      ISSGGEERLASHNLFREEEQCDLPKISQLDGVDDGTESDTSVTATTRKSSQIPKRNGKENGTENLKIDRPEDAGEKEHVI
H_KMT2A      ISSGGEERLASHNLFREEEQCDLPKISQLDGVDDGTESDTSVTATTRKSSQIPKRNGKENGTENLKIDRPEDAGEKEHVI
WH_KMT2A     ISSGGEERLASHNLFREEEQCDLPKISQLDGVDDGTESDTSVTATTRKSSQIPKRNGKENGTENLKIDRPEDAGEKEHVI
M_KMT2A      ISSGGEERLASHNLFREEEQCDLPKISQLDGVDDGTESDTSVTATSRKSSQIPKRNGKENGTENLKIDRPEDAGEKEHVI
H_KMT2A-1    ISSGGEERLASHNLFREEEQCDLPKISQLDGVDDGTESDTSVTATTRKSSQIPKRNGKENGTENLKIDRPEDAGEKEHVT
H_KMT2A-2    ISSGGEERLASHNLFREEEQCDLPKISQLDGVDDGTESDTSVTATTRKSSQIPKRNGKENGTENLKIDRPEDAGEKEHVT
P_KMT2A      IASGGEERLASHNLFREEEQCDLPKISQLDGVDDGTESDTSVTATTRKSSQIPKRNSKENGTENLKMDRPEDAGEKEHVI

                     1050      1060      1070      1080      1090      1100      1110      1120
             ....|....|....|....|....|....|....|....|....|....|....|....|....|....|....|....|
D_KMT2A      KSSVGHKNEPKMDNCHSVSRVKTQGQDSLEAQLSSLESSRRVHTSTPSDKNLLDTYNPELLKSDSDNNNSDDCGNILPSD
H_KMT2A      KSSVGHKNEPKMDNCHSVSRVKTQGQDSLEAQLSSLESSRRVHTSTPSDKNLLDTYNPELLKSDSDNNNSDDCGNILPSD
WH_KMT2A     KSSVGHKNEPKMDNCHSVSRVKTQGQDSLEAQLSSLESSRRVHTSTPSDKNLLDTYNPELLKSDSDNNNSDDCGNILPSD
M_KMT2A      KSAVGHKNEPKLDNCHSVSRVKAQGQDSLEAQLSSLESSRRVHTSTPSDKNLLDTYNAELLKSDSDNNNSDDCGNILPSD
H_KMT2A-1    KSSVGHKNEPKMDNCHSVSRVKTQGQDSLEAQLSSLESSRRVHTSTPSDKNLLDTYNTELLKSDSDNNNSDDCGNILPSD
H_KMT2A-2    KSSVGHKNEPKMDNCHSVSRVKTQGQDSLEAQLSSLESSRRVHTSTPSDKNLLDTYNTELLKSDSDNNNSDDCGNILPSD
P_KMT2A      KSSVGHKNEPKMDNCHSVSRVKTQGQDSLEAQLSSLESGRRVHTSTPSDKNLLDTYNTELLKSDSDNNNSDDCGNILPSD

                     1130      1140      1150      1160      1170      1180      1190      1200
             ....|....|....|....|....|....|....|....|....|....|....|....|....|....|....|....|
D_KMT2A      IMDFVLKNTPSMQALGESPESSSSELLNLGEGLGLDSNREKDMGLFEVFSQQLPPTEPVDSSVSSSISAEEQFELPLELP
H_KMT2A      IMDFVLKNTPSMQALGESPESSSSELLNLGEGLGLDGNREKDMGLFEVFSQQLPPTEPVDSSVSSSISAEEQFELPLELP
WH_KMT2A     IMDFVLKNTPSMQALGESPESSSSELLNLGEGLGLDGNREKDMGLFEVFSQQLPPTEPVDSSVSSSISAEEQFELPLELP
M_KMT2A      IMDFVLKNTPSMQALGESPESSSSELLTLGEGLGLDSNREKDIGLFEVFSQQLPATEPVDSSVSSSISAEEQFELPLELP
H_KMT2A-1    IMDFVLKNTPSMQALGESPESSSSELLNLGEGLGLDSNREKDMGLFEVFSQQLPTTEPVDSSVSSSISAEEQFELPLELP
H_KMT2A-2    IMDFVLKNTPSMQALGESPESSSSELLNLGEGLGLDSNREKDMGLFEVFSQQLPTTEPVDSSVSSSISAEEQFELPLELP
P_KMT2A      IMDFVLKNTPSMQALGESPESSSSELLNLGEGLGLDSNREKDMGLFEVFSQQLPTAEPVDSSVSSSISAEEQFELPLELP
```

```
                   1210      1220      1230      1240      1250      1260      1270      1280
           ....|....|....|....|....|....|....|....|....|....|....|....|....|....|....|....|
D_KMT2A    SDLSVLTTRSPTVPSQNPSRLAVISDSGEKRVTITEKSVASSEGDSALLSPGVDPTPEGHMTPDHFIQGHMDADHISSPP
H_KMT2A    SDLSVLTTRSPTVPSQNPSRLAVISDSGEKRVTITEKSVASSEGDSALLSPGVDPTPEGHMTPDHFIQGHMDADHISSPP
WH_KMT2A   SDLSVLTTRSPTVPSQNPSRLAVISDSGEKRVTITEKSVASSEGDSALLSPGVDPTPEGHMTPDHFIQGHMDADHISSPP
M_KMT2A    SDLSVLTTRSPTVPSQNPSRLAVISDSGEKRVTITEKSVASSEGDPALLSPGVDPAPEGHMTPDHFIQGHMDADHISSPP
H_KMT2A-1  SDLSVLTTRSPTVPSQNPSRLAVISDSGEKRVTITEKSVASSESDPALLSPGVDPTPEGHMTPDHFIQGHMDADHISSPP
H_KMT2A-2  SDLSVLTTRSPTVPSQNPSRLAVISDSGEKRVTITEKSVASSESDPALLSPGVDPTPEGHMTPDHFIQGHMDADHISSPP
P_KMT2A    SDLSVLTTRSPTVPSQNPSRLAVISDSGEKRVSITEKSVASSESDSALLSPGVDPTPEGHMTPDHFIQGHMDADHISSPP

                   1290      1300      1310      1320      1330      1340      1350      1360
           ....|....|....|....|....|....|....|....|....|....|....|....|....|....|....|....|
D_KMT2A    CGSVEQGHGNNQDLTRNSSTPGLQVPVSPTVPIQNQKYVPNSTDSPGPSQISNAAVQTTPPHLKPATEKLIVVNQNMQPL
H_KMT2A    CGSVEQGHGNNQDLTRNSSTPGLQA-------------------------------------------------------
WH_KMT2A   CGSVEQGHGNNQDLTRNSSTPGLQVPVSPTVPIQNQKYVPNSTDSPGPSQISNAAVQTTPPHLKPATEKLIVVNQNMQPL
M_KMT2A    CGSVEQGHGNSQDLTRNSGTPGLQVPVSPTVPVQNQKYVPSSTDSPGPSQISNAAVQTTPPHLKPATEKLIVVNQNMQPL
H_KMT2A-1  CGSVEQGHGNNQDLTRNSSTPGLQVPVSPTVPIQNQKYVPNSTDSPGPSQISNAAVQTTPPHLKPATEKLIVVNQNMQPL
H_KMT2A-2  CGSVEQGHGNNQDLTRNSSTPGLQVPVSPTVPIQNQKYVPNSTDSPGPSQISNAAVQTTPPHLKPATEKLIVVNQNMQPL
P_KMT2A    CGSVEQGHGNNQDLTRNSSTPGLQVPVSPTVPIQNQKYVPNSTDSPGPSQISNAAVQTTPPHLKPATEKLIVVNQNMQPL

                   1370      1380      1390      1400      1410      1420      1430      1440
           ....|....|....|....|....|....|....|....|....|....|....|....|....|....|....|....|
D_KMT2A    YVLQTLPNGVTQKIQLTSSVSSTPNVMETNTSVLGPMGGGLTLTTGLNPSLPTSQPLFPPASKGLLPMPHHQHLHSFPAA
H_KMT2A    --------------------------------------------------------------------------------
WH_KMT2A   YVLQTLPNGVTQKIQLTSSVSSTPNVMETNTSVLGPMGGGLTLTTGLNPSLPTSQPLFPPASKGLLPMPHHQHLHSFPAA
M_KMT2A    YVLQTLPNGVTQKIQLTSPVSSTPSVMETNTSVLGPMGSGLTLTTGLNPSLPPSPSLFPPASKGLLSVPHHQHLHSFPAA
H_KMT2A-1  YVLQTLPNGVTQKIQLTSSVSSTPSVMETNTSVLGPMGGGLTLTTGLNPSLPTSQSLFPSASKGLLPMSHHQHLHSFPAA
H_KMT2A-2  YVLQTLPNGVTQKIQLTSSVSSTPSVMETNTSVLGPMGGGLTLTTGLNPSLPTSQSLFPSASKGLLPMSHHQHLHSFPAA
P_KMT2A    YVLQTLPNGVTQKIQLTSSVSSAPNVMETNTSVLGPMGSGLTLTTGLNPSLPTSQSLFPPASKGLLPMPHHQHLHSFPAA

                   1450      1460      1470      1480      1490      1500      1510      1520
           ....|....|....|....|....|....|....|....|....|....|....|....|....|....|....|....|
D_KMT2A    TQSSFPPNISSPPSGLLIGVQPPPDPQLLVSEASQRTDLSTTVATPSSGLKKRPISRLQTRKNKKLAPSSTPSNIAPSDV
H_KMT2A    --------------------------------------------------------------------------------
WH_KMT2A   TQSSFPPNISSPPSGLLIGVQPPPDPQLLVSEASQRTDLSTTVATPSSGLKKRPISRLQTRKNKKLAPSSTPSNIAPSDV
M_KMT2A    AQSSFPPNISSPPSGLLIGVQPPPDPQLLGSEANQRTDLTTTVATPSSGLKKRPISRLHTRKNKKLAPSSAPSNIAPSDV
H_KMT2A-1  TQSSFPPNISNPPSGLLIGVQPPPDPQLLVSESSQRTDLSTTVATPSSGLKKRPISRLQTRKNKKLAPSSTPSNIAPSDV
H_KMT2A-2  TQSSFPPNISNPPSGLLIGVQPPPDPQLLVSESSQRTDLSTTVATPSSGLKKRPISRLQTRKNKKLAPSSTPSNIAPSDV
P_KMT2A    TQSSFPTNISSPPSGLLIGVQPPPDPQLLVSEASQRTDLSTTVATPPSGLKKRPISRLQNRKNKKLAPSSTPSNIAPSDV

                   1530      1540      1550      1560      1570      1580      1590      1600
           ....|....|....|....|....|....|....|....|....|....|....|....|....|....|....|....|
D_KMT2A    VSNMTLINFTPSQLPNHPNLLDLGSLNTSSHRTVPNIIKRSKSGIMYFEQAPLLP-QSVGGTAATAAGTSTISQDTGHLT
H_KMT2A    ---------------------------------------------------PLLP-QRVGGSAATAAGTSTISQDSGHLT
WH_KMT2A   VSNMTLINFTPSQLPNHPNLLDLGSLNTSSHRTVPNIIKRSKSGIMYFEQAPLLP-QRVGGSAATAAGTSTISQDSGHLT
M_KMT2A    VSNMTLINFTPSQLSNHPSLLDLGSLNPSSHRTVPNIIKRSKSGIMYFEQAPLLPPQSVGGTAATAAGSSTISQDTSHLT
H_KMT2A-1  VSNMTLINFTPSQLPNHPSLLDLGSLNTSSHRTVPNIIKRSKSSIMYFEPAPLLP-QSVGGTAATAAGTSTISQDTSHLT
H_KMT2A-2  VSNMTLINFTPSQLPNHPSLLDLGSLNTSSHRTVPNIIKRSKSSIMYFEPAPLLP-QSVGGTAATAAGTSTISQDTSHLT
P_KMT2A    VSNMTLINFTPSQLPNHPNLLDLGSLNASSHRTVPNIIKRSKSSIMYFEQAPLLP-QSVGGSAAPAAGTSTISQDAGHLT

                   1610      1620      1630      1640      1650      1660      1670      1680
           ....|....|....|....|....|....|....|....|....|....|....|....|....|....|....|....|
D_KMT2A    SGPVSGLASGSSVLNVVSMPTTTAPTSSASVPGHVALTNPRLLGAPDIGSISNLLIKASQQSLGIQDQPVALPPSSGMFP
H_KMT2A    SGPVSGLASGSSVLNVVSMPTTTAPASSASVPGHVALTNPRLLGAPDIGSISNLLIKASQQSLGIQDQPVALPPSSGMFP
WH_KMT2A   SGPVSGLASGSSVLNVVSMPTTTAPASSASVPGHVALTNPRLLGAPDIGSISNLLIKASQQSLGIQDQPVALPPSSGMFP
M_KMT2A    SGPVSALASGSSVLNVVSMQTTAAPTSSTSVPGHVTLANQRLLGTPDIGSISHLLIKASHQSLGIQDQPVALPPSSGMFP
H_KMT2A-1  SGSVSGLASSSSVLNVVSMQTTTTPTSSASVPGHVTLTNPRLLGTPDIGSISNLLIKASQQSLGIQDQPVALPPSSGMFP
H_KMT2A-2  SGSVSGLASSSSVLNVVSMQTTTTPTSSASVPGHVTLTNPRLLGTPDIGSISNLLIKASQQSLGIQDQPVALPPSSGMFP
P_KMT2A    SGPVSGLASGSSVLNVVSMQTTTAPTSSASVPGHVALTNPRLLGTPDIGSISNLLIKASQQSLGIQDQSVALPPSSGMFP

                   1690      1700      1710      1720      1730      1740      1750      1760
           ....|....|....|....|....|....|....|....|....|....|....|....|....|....|....|....|
D_KMT2A    QLGTSQTPSTAAMTAASSICVLPSTQTTGITAASPSGEAGEHYQLQHVNQLLASKTGLLSSQRD--LDSAPGTQGSNYTQ
H_KMT2A    QLGTSQTPSTAAMTAASSICVLPSTQTTGITAASPSGEAGEHYQLQHVNQLLASKTGLLSSQRD--LDSAPGTQGSNFTQ
WH_KMT2A   QLGTSQTPSTAAMTAASSICVLPSTQTTGITAASPSGEAGEHYQLQHVNQLLASKTGLLSSQRD--LDSAPGTQGSNFTQ
M_KMT2A    QLGTSQTPSAAAMTAASSICVLPSSQTAGMTAASPPGEAEEHYKLQRGNQLLAGKTGTLTSQRDRDPDSAPGTQPSNFTQ
H_KMT2A-1  QLGTSQTPSTAAITAASSICVLPSTQTTGITAASPSGEADEHYQLQHVNQLLASKTGIHSSQRD--LDSASGPQVSNFTQ
H_KMT2A-2  QLGTSQTPSTAAITAASSICVLPSTQTTGITAASPSGEADEHYQLQHVNQLLASKTGIHSSQRD--LDSASGPQVSNFTQ
P_KMT2A    QLGTSQTPSTAAMTAASSICVLPSTQTAGITAASPSGDTDEHYQLQHVNQLLASKTGILSSQRD--LDSAPGTQGSNFTQ

                   1770      1780      1790      1800      1810      1820      1830      1840
           ....|....|....|....|....|....|....|....|....|....|....|....|....|....|....|....|
D_KMT2A    TVDAPNSMGLEQNKALSSAMQASSASPGGSPSS---GQQSASPSVPGPTKPKPKIKRIQLPLDKGNGKKHKVSHLRTSS-
H_KMT2A    TVDAPNSMGLEQNKALSSAMQASSASPGGSPSS---GQQSASPSVPGPTKPKPKIKRIQLPLDKGNGKKHKVSHLRTSS-
WH_KMT2A   TVDAPNSMGLEQNKALSX-------SPGGSPSS---GQQSASPSVPGPTKPKPKIKRIQLPLDKGNGKKHKVSHLRTSS-
```

```
M_KMT2A      TAEAPNGVRLEQNKTLPSAKPASSASPGSSPSS---GQQSGSSSVPGPTKPKPKAKRIQLPLDKGSGKKHKVSHLRTSS-
H_KMT2A-1    TVDAPNSMGLEQNKALSSAVQASPTSPGGSPSSPSSGQRSASPSVPGPTKPKPKTKRFQLPLDKGNGKKHKVSHLRTSSS
H_KMT2A-2    TVDAPNSMGLEQNKALSSAVQASPTSPGGSPSSPSSGQRSASPSVPGPTKPKPKTKRFQLPLDKGNGKKHKVSHLRTSSS
P_KMT2A      TVDAPNNMGLEQNKTLSSAMQASSTSPGGSPSS---GQQSASPSVPAPTKPKPKSKRIQLPLDKGSGKKHKVSHLRTSS-

                       1850      1860      1870      1880      1890      1900      1910      1920
             ....|....|....|....|....|....|....|....|....|....|....|....|....|....|....|....|
D_KMT2A      EAHIPDQEANTTPLTSVTGTPGAEPEQQDTANVEQSSQKECGQPAGQVAALPEIQMTQNPTNEQESTEPKTVEEEESNFS
H_KMT2A      EAHIPDQEANTTPLTSVTGTPGAEPEQQDTANVEQSSQKECGQPAGQVAALPEIQMTQNPANEQESTEPKTVEEEESNFS
WH_KMT2A     EAHIPDQEANTTPLTSVTGTPGAEPEQQDTANVEQSSQKECGQPAGQVAALPEIQMTQNPANEQESTEPKTVEEEESNFS
M_KMT2A      EAHIPHRDTDPAPQPSVTRTPRANREQQDAAGVEQPSQKECGQPAGPVAALPEVQATQNPANEQENAEPKAMEEEESGFS
H_KMT2A-1    EAHIPDQ--ETTSLTSGTGTPGAEAEQQDTASVEQSSQKECGQPAGQVAVLPEVQVTQNPANEQESAEPKTVEEEESNFS
H_KMT2A-2    EAHIPDQ--ETTSLTSGTGTPGAEAEQQDTASVEQSSQKECGQPAGQVAVLPEVQVTQNPANEQESAEPKTVEEEESNFS
P_KMT2A      EAHIPDQEASTTALTSVTGTPGAEAEQQETASVEQSSQKGCGQPAGQVAVLPEIQTTQNPVNEQENSEPKTAEEEESNFS

                       1930      1940      1950      1960      1970      1980      1990      2000
             ....|....|....|....|....|....|....|....|....|....|....|....|....|....|....|....|
D_KMT2A      SPLMLWLQQEQKRKESIAEKKPKKGLVFEISSDDGFQICAESIEDAWKSLTDKVQEARSNARLKQLSFAGVNGLRMLGIL
H_KMT2A      SPLMLWLQQEQKRKESIAEKKPKKGLVFEISSDDGFQICAESIEDAWKSLTDKVQEARSNARLKQLSFAGVNGLRMLGIL
WH_KMT2A     SPLMLWLQQEQKRKESIAEKKPKKGLVFEISSDDGFQICAESIEDAWKSLTDKVQEARSNARLKQLSFAGVNGLRMLGIL
M_KMT2A      SPLMLWLQQEQKRKESITERKPKKGLVFEISSDDGFQICAESIEDAWKSLTDKVQEARSNARLKQLSFAGVNGLRMLGIL
H_KMT2A-1    SPLMLWLQQEQKRKESITEKKPKKGLVFEISSDDGFQICAESIEDAWKSLTDKVQEARSNARLKQLSFAGVNGLRMLGIL
H_KMT2A-2    SPLMLWLQQEQKRKESITEKKPKKGLVFEISSDDGFQICAESIEDAWKSLTDKVQEARSNARLKQLSFAGVNGLRMLGIL
P_KMT2A      SPLMLWLQQEQKRKESIAEKKPKKGLVFEISSDDGFQICAESIEDAWKSLTDKVQEARSNARLKQLSFAGVNGLRMLGIL

                       2010      2020      2030      2040      2050      2060      2070      2080
             ....|....|....|....|....|....|....|....|....|....|....|....|....|....|....|....|
D_KMT2A      HDAVVFLIEQLSGAKHCRNYKFRFHKPEEANEPPLNPHGSARAEVHLRQVPFFSISGLVSUFYYLMIKLFSHVTFEISNV
H_KMT2A      HDAVVFLIEQLSGAKHCRNYKFRFHKPEEANEPPLNPHGSARAEVHLRKS------------------------------
WH_KMT2A     HDAVVFLIEQLSGAKHCRNYKFRFHKPEEANEPPLNPHGSARAEVHLRKS------------------------------
M_KMT2A      HDAVVFLIEQLAGAKHCRNYKFRFHKPEEANEPPLNPHGSARAEVHLRQS------------------------------
H_KMT2A-1    HDAVVFLIEQLSGAKHCRNYKFRFHKPEEANEPPLNPHGSARAEVHLRKS------------------------------
H_KMT2A-2    HDAVVFLIEQLSGAKHCRNYKFRFHKPEEANEPPLNPHGSARAEVHLRKS------------------------------
P_KMT2A      HDAVVFLIEQLSGAKHCRNYKFRFHKPEEANEPPLNPHGSARAEVHLRQS------------------------------

                       2090      2100      2110      2120      2130      2140      2150      2160
             ....|....|....|....|....|....|....|....|....|....|....|....|....|....|....|....|
D_KMT2A      NPILRCFCUSISLNIFUSFGSRKSAFDMFNFLASKHRQPPEYNPNDEEEEEVQLKSARRATSMDLPMPMRFRHLKKTSKE
H_KMT2A      ------------------------AFDMFNFLASKHRQPPEYNPNDEEEEEVQLKSARRATSMDLPMPMRFRHLKKTSKE
WH_KMT2A     ------------------------AFDMFNFLASKHRQPPEYNPNDEEEEEVQLKSARRATSMDLPMPMRFRHLKKTSKE
M_KMT2A      ------------------------AFDMFNFLASKHRQPPEYNPNDEEEEEVQLKSARRATSMDLPMPMRFRHLKKTSKE
H_KMT2A-1    ------------------------AFDMFNFLASKHRQPPEYNPNDEEEEEVQLKSARRATSMDLPMPMRFRHLKKTSKE
H_KMT2A-2    ------------------------AFDMFNFLASKHRQPPEYNPNDEEEEEVQLKSARRATSMDLPMPMRFRHLKKTSKE
P_KMT2A      ------------------------AFDMFNFLASKHRQPPEYNPNDEEEEEVQLKSARRATSMDLPMPMRFRHLKKTSKE

                       2170      2180      2190      2200      2210      2220      2230      2240
             ....|....|....|....|....|....|....|....|....|....|....|....|....|....|....|....|
D_KMT2A      AVGVYRSPIHGRGLFCKRNIDAGEMVIEYAGNVIRSIQTDKREKYYDSKGIGCYMFRIDDSEVVDATMHGNAARFINHSC
H_KMT2A      AVGVYRSPIHGRGLFCKRNIDAGEMVIEYAGNVIRSIQTDKREKYYDSKGIGCYMFRIDDSEVVDATMHGNAARFINHSC
WH_KMT2A     AVGVYRSPIHGRGLFCKRNIDAGEMVIEYAGNVIRSIQTDKREKYYDSKGIGCYMFRIDDSEVVDATMHGNAARFINHSC
M_KMT2A      AVGVYRSPIHGRGLFCKRNIDAGEMVIEYAGNVIRSIQTDKREKYYDSKGIGCYMFRIDDSEVVDATMHGNAARFINHSC
H_KMT2A-1    AVGVYRSPIHGRGLFCKRNIDAGEMVIEYAGNVIRSIQTDKREKYYDSKGIGCYMFRIDDSEVVDATMHGNAARFINHSC
H_KMT2A-2    AVGVYRSPIHGRGLFCKRNIDAGEMVIEYAGNVIRSIQTDKREKYYDSKGIGCYMFRIDDSEVVDATMHGNAARFINHSC
P_KMT2A      AVGVYRSPIHGRGLFCKRNIDAGEMVIEYAGNVIRSIQTDKREKYYDSKGIGCYMFRIDDSEVVDATMHGNAARFINHSC

                       2250      2260      2270      2280      2290      2300
             ....|....|....|....|....|....|....|....|....|....|....|....|
D_KMT2A      EPNCYSRVINIDGQKHIVIFAMRKIYRGEELTYDYKFPIEDASNKLPCNCGAKKCRKFLN
H_KMT2A      EPNCYSRVINIDGQKHIVIFAMRKIYRGEELTYDYKFPIEDASNKLPCNCGAKKCRKFLN
WH_KMT2A     EPNCYSRVINIDGQKHIVIFAMRKIYRGEELTYDYKFPIEDASNKLPCNCGAKKCRKFLN
M_KMT2A      EPNCYSRVINIDGQKHIVIFAMRKIYRGEELTYDYKFPIEDASNKLPCNCGAKKCRKFLN
H_KMT2A-1    EPNCYSRVINIDGQKHIVIFAMRKIYRGEELTYDYKFPIEDASNKLPCNCGAKKCRKFLN
H_KMT2A-2    EPNCYSRVINIDGQKHIVIFAMRKIYRGEELTYDYKFPIEDASNKLPCNCGAKKCRKFLN
P_KMT2A      EPNCYSRVINIDGQKHIVIFAMRKIYRGEELTYDYKFPIEDASNKLPCNCGAKKCRKFLN
```
